# Unspecified pain and other soft tissue disorders following electrical injuries: a register-based matched cohort study

**DOI:** 10.1007/s00420-021-01802-y

**Published:** 2021-10-10

**Authors:** Per Hoegh Poulsen, Ole Carstensen, Anette Kærgaard, Jesper Medom Vestergaard, Kent J. Nielsen, Karin Biering

**Affiliations:** Danish Ramazzini Centre, Department of Occupational Medicine, University Research Clinic, Regional Hospital West Jutland, Gl. Landevej 61, 7400 Herning, Denmark

**Keywords:** Electrical shock, Pain, Matched cohort study, Electrical injury

## Abstract

**Objective:**

This study investigates whether individuals who have sustained an electrical injury (EI) are diagnosed with unspecified pain or pain related to the musculoskeletal system in the years following the injury.

**Methods:**

Individuals listed in Danish registers as having sustained EIs were matched for sex, age, and year of injury in a cohort study with individuals having experienced dislocations/sprains (match 1), eye injuries (match 2), and a sample of individuals with the same occupation without a history of electrical injuries (match 3). Outcomes were *unspecified pain* and *unspecified soft tissue disorders*. Conditional logistic regression and conditional Cox regression were applied.

**Results:**

We identified 14,112 individuals who sustained EIs. A higher risk of both outcomes was observed for all three matches, and was highest at the 6- and 12-month follow-ups. The risk of both outcomes was considerably higher for match 3.

**Conclusions:**

This study confirms that exposure to EIs increases the risk of being diagnosed with *unspecified pain* or *unspecified soft tissue disorders* both at short and long terms. Our results also showed that the risk of unspecified pain as sequelae is related to the severity of the injury.

## Introduction

People of all ages are exposed to electric shocks, for example, at home or at work. Most people who are exposed to electric shocks experience brief pain and discomfort, for which they do not seek medical attention. However, in some cases electric shocks cause injuries by damaging tissue as the electrical current passes through the body. The severity of such electrical injuries (EIs) depends on several factors, such as voltage, duration, and being stuck to the power-source, and ranges from immediate internal or external physiological effects (tissue damage or burns), to heart failure, and in rare cases, death (Arnoldo et al. 2004; Duff and McCaffrey 2001; Koumbourlis 2002; Wesner and Hickie 2013). Furthermore, some of the people who sustain an EI experience long-term sequela, such as pain, paraesthesia, and sensory and motor deficits (Wesner and Hickie 2013). For example, a retrospective Swedish study of professional electricians showed that about 10% of the study population reported persistent symptoms between 1 and 45 years after an initial injury. The significant persistent symptoms were pain, loss of sensation, and/or muscle weakness (Rådman et al. 2016). Pain has also been observed as a significant long-term sequela of EIs in other studies (Bailey et al. 2008; Chudasama et al. 2010; Morse and Morse 2005; Piotrowski et al. 2014).

Pain as a long-term or delayed physiological sequela after EIs may sometimes be overlooked in patients, especially after EIs with less severe immediate physiological symptoms. This group of patients presents with various forms of pain that are very difficult for the physician to relate to a specific clinical diagnosis. The results of a study by Fish et al. showed that patients that had sustained low-voltage EIs are often referred for specialized consultations and tests, which generally are ineffective for correlating their long-term symptoms with the initial EI (Fish et al. 2012). This may be because sequela of EIs resemble those of many other conditions, therefore specialists perform examinations that cannot pick up the low-level damage that has occurred (Fish et al. 2012). Thus, these patients are often examined by various specialists in various medical specialities, and end up with diagnoses of unspecified (pain), owing to the absence of objective findings and positive clinical tests. A study that re-evaluated the long-term complications in 53 electrical shock patients found that more than 95% of the referring physicians’ diagnoses, such as chronic pain of unknown aetiology, or pain in an arm or leg, could not be confirmed by a re-evaluation. The author claims this could be due to many of the clinical symptoms are not well-recognized by the medical community as sequela of electrical shocks (Hendler 2005).

To the best of our knowledge, as yet there is no literature that specifically examines pain as a long-term sequela of EIs. This may be because pain is usually part of other diagnoses, for example, when examining peripheral nerve diseases or musculoskeletal sequela.

This study investigates whether individuals who have sustained EIs are diagnosed with unspecified pain or pain related to the musculoskeletal system in the years following the injury.

We hypothesize that individuals exposed to EIs have a higher risk of being diagnosed with unspecified pain or pain related to the musculoskeletal system compared to matched controls.

## Materials and methods

### Materials

This study is a matched cohort study based on EIs documented in two population-based registers, the Danish National Patient Register (DNPR) and the register of reported occupational injuries of the Danish Working Environment Authority (DWEA). It also includes data from other population-based registers in Statistics Denmark, described in detail in the next section. This study is part of a large matched cohort study that aims to examine the associations between EIs and neurologic, psychological, and physiological sequela (Biering et al. 2021a, b).

The DNPR comprises all hospital contacts in Denmark, including information on injuries, diagnoses, procedures for in- and outpatients, and emergency visits (Lynge et al. 2011; Schmidt et al. 2015). The DWEA register includes occupational injuries reported by employers, employees, unions, and healthcare workers. Danish employers must report any work-related injury that results in sick leave "for one or more days" following that of the injury. The DWEA register is primarily intended for keeping track of occupational injuries (European Agency for Safety and Health at Work 2021).

### Methods

This study included Danish EIs reported to either the DNPR (1994–2016) or the DWEA register (2005–2016). Although DNPR was established in 1977, information about EIs was not adequately registered before the International Statistical Classification of Diseases and Related Health Problems, 10th Revision (ICD-10) was established in 1994. The DNPR and the DWEA register were combined to identify as many cases of EIs as possible. We decided to include cases of EIs from 1996 to 2014 in this study. We made this decision to allow for at least 2 years of time clear of the outcomes of interest before the EI, and at least 2 years for the outcomes to occur after the EI. If identified in the DWEA register the injury was an occupational injury, this was not necessarily the case if the injury was identified in the DNPR. Underreporting is a well-known problem in the DWEA register (Lander et al. 2014, 2015).

### Participants

In the DNPR individuals with hospital visits as the result of EI were identified by selecting contacts coded with the ICD-10 classification, DT754 (electrocution), and the Danish mechanisms of injury classifications, EUHA10 (Release of electrical energy), EUYD4 (Electrical installations/systems), EUWA10 (Self-harm act with electrical energy), or EUYZ0020 (Electrical current). The DT754 code was applied to the entire period studied, whereas the injury codes (EU*) were applied only to instances from 2000 and on, when a separate injury register was established. Injured individuals seen by casualty departments, admitted to hospitals, and in outpatient clinics were included. The DWEA register identified individuals with EIs according to information for two types of exposure: ‘Acute/brief exposure to welding arc or electric arc’ or ‘Acute/brief exposure to electricity or reception of electric charge in the body’. To avoid duplicate analyses of any one accident because of reports to both registers with a small difference in dates, only accidents registered with the DWEA register were included in our study. Only the first injury for each individual was included, regardless of cause. Injury records from the DNPR and the DWEA register were linked to Statistics Denmark, using a unique identification number (CPR number) and injury dates/year. Every Danish citizen as well as registered migrant workers holds this number that provide the possibility to link each person across different registries (Schmidt et al. 2014). Statistics Denmark is the central authority for Danish registries and statistics. We used the following registers in this study, the population register (Statistics Denmark 2021b) (to determine sex and age at time of matching), the Register-based Labour Force Statistics (RAS) register (Register-based Labour Force Statistics 2020) (to determine whether a participant was part of the workforce at the time of the injury, and to identify matched controls from the workforce), the migration register (Statistics Denmark 2021c) (to derive date for possible migration) and the register of deaths (Statistics Denmark 2021a) (to derive date for death).

For our sensitivity analysis, we accessed information about lengths of hospital stays from the DNPR. Length of hospitalization, including time spent in the casualty department, was determined for all hospital admissions. We used this as a proxy for an injury's severity, working with the premise that the most severe injuries would also result in the longest hospital stays. Not all injuries in the DWEA register could be assigned a length of hospitalization, as in some cases we could not identify any hospital contact at the time of the injury.

### Matching

Every individual included in our study was matched in three different ways with others from the same data source (the DNPR or the DWEA register). We decided to make three different matches, because the perfect match controls were difficult to define. An injury had to be common for us to find a sufficient number of match controls, and it had to be unrelated to the outcomes in which we were interested. Therefore, we identified match controls as described below. We also matched controls with the same occupation. We excluded individuals who did not match with at least one other. Match controls could be used more than once, and individuals with an EI could be used as controls before they sustained their EI.

**Match 1** Injury match, dislocations/sprains: Individuals who had sustained an EI were matched with up to ten others with dislocations/sprains (‘DS93’ in the DNPR, and ‘sprains’ in the DWEA register).

**Match 2** Injury match, eye: Individuals who had sustained an EI were matched with up to ten others with an eye injury (‘DT15’ in the DNPR). No eye injuries were identified by the DWEA register, owing to missing information about eye injuries.

The matching variables were sex, age, and year of injury. The last was included to take changes in registration practise into account. For all matches, the match controls were randomly chosen if more than ten controls were available per individual, who sustained an EI. This randomizing made it possible for the same subject to act as a match control for more than one EI, though for only one particular year. If it was not possible to match an individual’s exact age, the algorithm identified the same-sex control closest in age in the same 5-year age group, and with the year of injury.

**Match 3** Occupation match: Individuals who had sustained an EI were matched with up to ten others from the workforce, from the same occupational group, sex, and age in the year of the EI. The injured individuals and the match controls were those who were employed at the time of the match, but the EI reported to the DNPR may have happened away from work. We assigned injury dates to match controls based on their match-individual’s injury, to identify outcomes before and after a specific point in time. The purpose of this match was to account for the fact that individuals employed in certain occupations may have a greater risk of the outcomes because of socioeconomic factors or occupational exposures other than exposure to EI.

### Outcomes

The outcomes of interest for ICD-10 medical diagnoses were *pain, unspecified* (R52) (hereafter, *unspecified pain*), and *other and unspecified soft tissue disorders, not elsewhere classified* (M79, M79.0, M79.1, M79.2, M79.6, M79.8, M79.9) (hereafter, *unspecified soft tissue disorders*).

If any of the exposed individuals or the potential match controls were registered with the outcome of interest before the matching process, they were excluded from the analysis. This was done separately for each outcome, to keep the individuals in the data set to the analysis of the other outcome. If individuals who sustained EI were excluded, all their matching controls were also excluded, whereas match controls were excluded individually, which kept the remaining match controls and the exposed individuals in the data set. This meant that the study sample was different for each analysis of the two outcomes.

For the occupation match, all individuals with an injury registered with the DWEA register were defined as part of the workforce, as their injuries occurred at work. However, Statistics Denmark did not define them all as part of the workforce, probably because some had sustained an EI while employed part-time (students, interns, or retired individuals). Thus, 175 individuals with an injury registered with the DWEA register could not be matched for the occupation match. They could be matched only for the injury match, with dislocations/sprains as controls.

Occupation was derived from the RAS register at Statistics Denmark using DISCO codes. DISCO is the official Danish version of International Standard Classifications of Occupations (ISCO), established by the International Labour Organization (ILO) (International Labour Organization 2020; available at https://www.ilo.org/public/english/bureau/stat/isco/). For matching groups, we applied the second level (two-digit) in the hierarchy. Current work status was also derived from the RAS register, to identify controls in the workforce.

Both individuals who sustained EIs and controls who emigrated or died during follow-up were censored from that date forward, as follow-up in the DNPR was impossible.

Registering accidents with the DNPR became mandatory in 2000. Before that, the accident code (DT754) was occasionally used, but not necessarily, if the main problem immediately following from the accident was something else, such as a burn or unconsciousness. From the DWEA register, data were available in Statistics Denmark in the period from 2005 to 2016, both years included.

All individuals who sustained an EI were followed for at least 2 years after the injury and up to 19 years after the injury (full follow-up). The majority of individuals (80%) were followed for minimum 4 years and 71% were followed for 5 years.

### Statistical methods

We compared the two matching groups using conditional logistic regression, where each match group consisted of one injured individual and up to ten match controls, depending on availability, and exclusions for each outcome. We also conducted a conditional Cox regression, to examine the outcomes in a time to event setting, to determine whether any of the outcomes occurred earlier for individuals who had sustained an EI. Schoenfeld’s residuals test was applied to confirm the proportional hazard.

The injuries we studied were a combination of occupational injuries and injuries in other settings. The DNPR did not provide information about the settings, but we tried to compensate for this with an additional analysis of the data set matched by injury, including only individuals in the workforce at the time of the injury.

We performed a number of additional analyses with EI individuals compared to match 1 controls. The three matches were comparable and thus only presented in the match 1 variant.

To identify possible gender differences, we carried out a supplementary analysis on time to event. To investigate possible late onset effects of the EI, we carried out a supplementary analysis, where for each follow-up we excluded individuals who reported the outcome before each follow-up period. Our sensitivity analysis excluded individuals who were hospitalized for less than 1 day, to determine whether more severe accidents revealed stronger associations between EIs and the two outcomes.

All procedures performed in this study were consistent with Danish ethical standards and with the Helsinki Declaration. The Regional Data Protection Agency approved this study (reference number 1-16-02-113-18). According to Danish law, register-based studies only need approval from the ethics committee if the data include human biological material (§ 14 in ‘Promulgation of the Act on the ethical treatment of health science research projects and health data science research projects’, available in Danish at www.retsinformation.dk/eli/lta/2020/1338). All data were stored and processed in a secure, protected server at Statistics Denmark. Results from Statistics Denmark are available to the researcher at the individual patient level, results may, however, only be presented at an aggregated level.

## Results

**Fig. 1:**
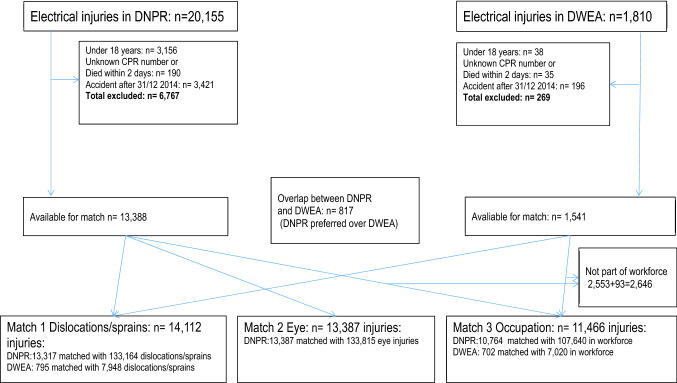
Descriptions of electrical injuries addressed in this study in the Danish National Patient Registry (DNPR) and the Danish Working Environment Authority (DWEA), and matches 1 to 3

The DNPR identified 20,155 EIs, and the DWEA register, 1,810 (Fig. 1 and Table 1, previously published in Biering et al. (2021a, b), available at https://oem.bmj.com/content/78/1/54). After we excluded people under the age of 18, individuals without a valid Danish identification number (CPR number), and individuals who died in the first 2 days following an EI, there was an overlap of 817 individuals from the two registers. Invalid identification numbers could indicate tourists or migrant workers with a temporary identification number, or simply mistyping in the DWEA register. When the overlap was resolved and only the initial EI was kept for each individual, we had 13,317 EIs from the DNPR and 795 EIs from the DWEA register for the injury matches. For the occupation match we also excluded 2646 individuals who were not in the workforce. We prioritized the entries in the DWEA register if there was a double registration, and this yielded 10,764 EIs from the DNPR and 702 EIs from the DWEA register, which were available for the occupation match. A match with 10 match controls was feasible for almost all EIs (Fig. 1).

**Table 1:** Description of the cohort with electrical injuries from the Danish National Patient Registry (DNPR) and the Danish Working Environment Authority (DWEA register), (*N* = 14,112)

	DNPR	DWEA^a^
*N*/%	*N* = 13,317	*N* = 795
Men (*n*/%)	10,180 (76.4%)	679 (85.4%)
Age groups (*n*/%)		
18–24	3884 (29.2%)	138 (17.4%)
25–29	2392 (18.0%)	109 (13.7%)
30–34	2024 (15.2%)	122 (15.5%)
35–39	1649 (12.4%)	110 (13.8%)
40–44	1163 (8.7%)	97 (12.2%)
45–49	875 (6.6%)	85 (10.7%)
50–54	628 (4.7%)	61 (7.7%)
55–59	390 (2.9%)	49 (6.2%)
60 +	312 (2.3%)	24 (3.0%)
Employed during the year of injury, by occupation (n/%)	10,764 (80.8%)	702 (88.3%)
1. Legislators, senior officials and managers/*Managers*	141 (1.1%)	< 5 (< 1%)
2. Professionals	962 (7.2%)	24 (3.0%)
3. Technicians and associate professionals	1047 (7.9%)	45 (5.7%)
4. Clerks/clerical support workers	408 (3.1%)	15 (1.9%)
5. Service workers and shop and market sales workers/*Services and Sales Workers*	1509 (11.3%)	70 (8.8%)
6. Skilled agricultural and fishery workers/*Skilled Agricultural, Forestry and Fishery Workers*	37 (0.3%)	< 5 (< 1%)
7. Craft and related trades workers	4914 (36.9%)	398 (50.1%)
8. Plant and machine operators and assemblers	631 (4.7%)	76 (9.6%)
9. Elementary occupations	991 (7.4%)	67 (8.4%)
10. Armed forces	124 (0.9%)	< 5 (< 1%)
Accident year (*n*/%)		
1996–1999	1368 (10.3%)	
2000–2004	2308 (17.3%)	
2005–2009	3677 (27.6%)	477 (60%)
2010–2014	5964 (44.8%)	318 (40%)
Hospitalization		
Less than 1 day	9,045 (67.9%)	193 (24.3%)
One day or more	2916 (21.9%)	154 (19.4%)
Missing/outpatient	1356 (10.2%)	448 (56.4%)
Died within 2 days from injury	6 (0.1%)	< 5 (< 1%)

^a^Overlap between registers removed, DNPR preferred over DWEA

The cohort is presented in Table 1. Men sustained the vast majority of the EIs, particularly those reported to the DWEA register, and younger individuals were overrepresented, which is most evident in injuries reported to the DNPR. The occupations with the highest incidence of EIs were craftworkers, however, service workers/sales staff were also overrepresented, even when compared to the distribution of occupations in Denmark. The length of hospitalization was most often less than a day. We observed that throughout the period studied, the number of EIs reported to the DNPR increased, whereas the number of EIs reported to the DWEA register decreased (Biering et al. 2021a, b).

For both outcomes, we excluded those who had been diagnosed with the outcome before they sustained the EI. The numbers of these exclusions are provided in Table 2. Frequencies of individuals with the various outcomes during the 5 years of follow-up and during the full follow-up are also presented in Table 2. In relation to match 1, we observed that 232 (1.6%) and 589 (4.2%) were excluded according to previous diagnosis, before they sustained an EI. During the 5 years of follow-up, 298 (2.1%) and 386 (2.7%) EI individuals were diagnosed with *unspecified pain* or *unspecified soft tissue disorders*, respectively. We also observed that 619 and 699 EI individuals were diagnosed with *unspecified pain* and *unspecified soft tissue disorder*, respectively, during full follow-up. Among these were 80 EI individuals given both diagnoses during full follow-up. Results were similar with regard to match 2. In general, with respect to match 3, we observed a smaller proportion who were excluded due to previous diagnosis and likewise with regard to those EI patients, who were diagnosed with the two outcomes during 5 years of follow-up and during full follow-up, compared to matches 1 and 2.

**Table 2:** Exclusions and diagnoses of electrical injured individuals for each of the three matches

Diagnoses	Match 1 Dislocations/sprains (14,112 injuries)			Match 2 Eye injuries (13,387 injuries)			Match 3 Occupation (11,466 injuries)		
	Excluded due to previous diagnosis	Diagnosed during 5 years	Diagnosed during full follow-up	Excluded due to previous diagnosis	Diagnosed during 5 years	Diagnosed during full follow-up	Excluded due to previous diagnosis	Diagnosed during 5 years	Diagnosed during full follow-up
	*n* (%)	*n* (%)	*n* (%)	*n* (%)	*n* (%)	*n* (%)	*n* (%)	*n* (%)	*n* (%)
Unspecified pain	232 (1.6)	298 (2.1)	619 (4.4)	222 (1.7)	288 (2.2)	601 (4.9)	162 (1.4)	205 (1.8)	456 (4.0)
Unspecified soft tissue disorders	589 (4.2)	386 (2.7)	699 (5.0)	568 (4.2)	370 (2.8)	672 (5.0)	430 (3.8)	290 (2.5)	536 (4.7)

Table 3 presents the main results of the associations between electrical injuries and the two outcomes as time to event and in different time periods over a range of 0–5 years.

**Table 3:** Associations between electrical injuries and unspecified pain and unspecified soft tissue disorders over the whole study and in time periods

Diagnoses	Match	Time to event	Time to event Workforce only	0–6 months	0–12 months	0–2 years	0–3 years	0–4 years	0–5 years
		HR	HR	OR	OR	OR	OR	OR	OR
		*n* = 14,112	*n* = 11,466	*n* = 14,064	*n* = 14,020	*n* = 13,936	*n* = 13,850	*n* = 13,786	*n* = 13,711
Unspecified pain	1 Dislocations/sprains	1.31 (1.20;1.43)	1.37 (1.23;1.52)	1.67 (1.09;2.40)	1.66 (1.26;2.19)	1.37 (1.12;1.68)	1.40 (1.19;1.64)	1.37 (1.19;1.58)	1.34 (1.18;1.52)
	2 Eye	1.32 (1.21;1.45)	1.36 (1.22;1.52)	1.45 (0.97;2.17)	1.47 (1.11;1.96)	1.37 (1.11;1.68)	1.47 (1.25;1.73)	1.48 (1.28;1.70)	1.44 (1.26;1.64)
	3 Occupation		1.84 (1.66;2.05)^	2.10 (1.26;3.49)	2.55 (1.78;3.64)	2.18 (1.70;2.80)	2.11 (1.73;2.59)	2.18 (1.84;2.59)	2.08 (1.78;2.43)
Unspecified soft tissue disorders	1 Dislocations/sprains	1.23 (1.13;1.33)	1.19 (1.08;1.31)	1.50 (1.11;2.05)	1.34 (1.07;1.67)	1.26 (1.07;1.48)	1.23 (1.07;1.41)	1.31 (1.16;1.48)	1.25 (1.12;1.40)
	2 Eye	1.32 (1.21;1.44)	1.29 (1.16;1.42)	1.79 (1.31;2.44)	1.58 (1.25;1.98)	1.49 (1.26;1.76)	1.42 (1.23;1.64)	1.52 (1.34;1.72)	1.47 (1.31;1.65)
	3 Occupation		1.85 (1.67;2.03)	2.40 (1.64;3.52)	2.36 (1.79;3.11)	2.22 (1.82;2.71)	2.16 (1.84;2.55)	2.23 (1.93;2.57)	2.09 (1.83;2.39)

*HR* hazard ratio, *OR* odds ratio, *n *number of injured individuals

^Proportional hazard not present

When we examined the association between EIs and *unspecified pain*, we found an increased risk of this outcome among EI individuals compared to all three matched groups. We observed that the risk of *unspecified pain* was highest at the 6- and 12-month follow-ups for EI's compared to match 1, whereas compared to match 2 the increased risk of *unspecified pain* among the EI individuals at 6 months was statistical insignificant. At 12 months and later, the risk was quite stable throughout the entire period studied. For match 3, the risk was highest at the 12-month follow-up and attenuated slightly in the subsequent follow-ups. We observed a tendency for the risk of *unspecified pain* to be considerably higher for EI individuals compared to match 3, with more than twice the risk even at the 5-year follow-up.

When we examined the association between EIs and *unspecified soft tissue disorders*, the picture was somewhat similar to that of *unspecified pain*. We found an increased risk of *unspecified soft tissue disorders* in all three matched groups. We observed that for all three matches, the overall risk was highest at 6 and 12 months after the EI. At the 2- and 3-year follow-ups on the injuries, the risk decreased a little, but appeared to increase slightly at the 4-year follow-up. The risk of *unspecified soft tissue disorders* was considerably higher when we matched with occupation controls, with over double the risk, even at the 5-year follow-up.

In general, all estimates were highest for match 3, where the match subjects were controls without an injury, but with the same occupation. When we restricted the analysis to the workforce only, this did not affect our estimates.

When the analyses were stratified by sex for match 1, this did not change the estimates with regard to *unspecified pain* and revealed only very limited difference with regard to *unspecified soft tissue disorders.*

When we restricted the analysis to individuals diagnosed with the outcome within each time interval (Table 4), our results showed that the risk was slightly reduced for both outcomes during the 5-year follow-up. The risk of *unspecified pain* was highest in the first year following an EI, however, there was still a greater than 30% higher risk of being diagnosed with *unspecified pain* 1 to 5 years later. The risk of *unspecified soft tissue disorders* was highest at 6-month follow-up, with a 20–30% higher risk of the outcome at the 4- and 5-year follow-ups.

**Table 4:** Associations between electrical injures and unspecified pain and unspecified soft tissue disorders for each time interval

Diagnoses	Match	0- < 6 months	6- < 12 months	1- < 2 years	2- < 3 years	3- < 4 years	4–5 years
		OR	OR	OR	OR	OR	OR
Unspecified pain		*n* = 31	*n* = 34	*n* = 58	*n* = 69	*n* = 60	*n* = 46
	1 Dislocations/sprains	1.62 (1.09;2.40)	1.69 (1.14;2.51)	1.30 (1.03;1.64)	1.36 (1.14;1.61)	1.34 (1.15;1.55)	1.31 (1.15;1.50)
Unspecified soft tissue disorders		*n* = 54	*n* = 46	*n* = 88	*n* = 74	*n* = 77	*n* = 47
	1 Dislocations/sprains	1.50 (1.11;2.05)	1.18 (0.85;1.63)	1.18 (0.97;1.43)	1.16 (1.00;1.37)	1.28 (1.12;1.46)	1.22 (1.08;1.38)

*OR* odds ratio

The sensitivity analysis (Table 5) revealed that when we restricted the analysis to hospitalizations of 1 day or longer, risk estimates of both outcomes increased considerably, especially for *unspecified pain*. This indicates that the risk of the two outcomes were related to the severity of the EI.

**Table 5:** Associations between electrical injuries (hospitalized for 1 day or more) and unspecified pain and unspecified soft tissue disorders

Diagnoses	Match	Time to event from Table 3	Time to event hospitalization > 1 day
		HR	HR
		*N* = 14,112	*N* = 3,070
Unspecified pain	1 Dislocations/sprains	1.31 (1.20;1.43)	1.73 (1.48;2.02)
Unspecified soft tissue disorders	1 Dislocations/sprains	1.23 (1.13;1.33)	1.43 (1.24;1.66)

*HR* hazard ratio

## Discussion

To the best of the authors’ knowledge, this is the first study using a matched design to examine the association between exposure to an EI and *unspecified pain* and *unspecified soft tissue disorders*. This study investigated 14,112 EIs from a 19-year period.

Our results showed a general pattern of people previously exposed to EIs having an increased risk of being diagnosed with *unspecified pain* or *unspecified soft tissue disorders* in the years following the EI, compared to matched controls. Thus, our findings support our main hypothesis.

For both diagnoses, we observed the strongest associations in the first 6 to 12 months following the EI, although this was not significant for *unspecified pain* for match 2 at 6 months. Overall, we observed the strongest associations in the occupation match (match 3), with more than twice the risk of both diagnoses during the 5-year follow-up. Our results also showed a higher risk of both diagnoses, when the analyses were restricted to new diagnosed patients in each time interval, indicating a greater risk of late onset effects, especially of *unspecified pain*. Furthermore, patients who had been hospitalized for more than 1 day had a considerably higher risk of being diagnosed with *unspecified pain*, compared to matched controls, which suggests that the risk of pain as a long-term sequela is also related to the severity of the EI.

In our sample, we observed that 298 (2.0%) and 386 (2.7%) persons were diagnosed with *unspecified pain* and *unspecified soft tissue disorders*, respectively, during a 5-year follow-up. Thus, a considerable number of patients in this study appear to experience long-term pain following an EI. We also observed that most patients were diagnosed with *unspecified pain* over a year after their EI. It is not possible to determine whether these findings are related to late onset of these symptoms, or because these patients had a long assessment process. However, we cannot dismiss the possibility of a late onset of pain, based on our data. In a study by Rådman et al., approximately 10% of electricians previously exposed to EIs reported persistent pain and muscular sequela 1 to 45 years after the injury (Rådman et al. 2016). Similarly, Bailey et al. (Bailey et al. 2008) found that pain as a long-term sequela was reported by 8% of the study population at the 1-year follow-up. Both studies applied self-reported information about pain related to electrical shock, which is prone to information bias. The outcomes discussed in our study were based on objective diagnoses, and, therefore, difficult to compare with the abovementioned studies. Nevertheless, in our data we noted a rather large group of patients that had a greater risk of unspecified pain, even after several years. This may indicate that people exposed to electrical current may in fact experience long-term pain, beyond symptoms that may be specifically diagnosed. Therefore, it appears that this group of patients needs further attention, and would perhaps benefit from a re-evaluation of their symptoms by a more specialized unit for various kinds of pain, as indicated in Hendler’s study (Hendler 2005).

The pathophysiological mechanisms of tissue damage following an EI are complex and very different from other traumatic injuries. So, whether these patients’ long-term pain is due to mechanisms triggered by the exposure to electrical shock, or perhaps because of limited ways of measuring this kind of symptoms, or is related to psychological factors, remains unanswered. This calls for further studies that may help to reveal the underlying mechanisms related to electrical shocks and pain, to facilitate and improve clinical praxis for this group of patients.

## Strengths and limitations

There were some limitations to this study. The registration of EIs in the DNPR was scanty in the early years of the study period. Probably only a small proportion of EIs were registered during the first years of this study. If the registered EIs differed in type, severity, or duration from the unregistered EIs, this could introduce bias in both directions. Underreporting of EIs may also be a more general problem in the DNPR, because detailed registration may not be prioritized in acute situations. If this is the case, the most severe injuries could have received an ICD-10 code, where the consequences of an EI, such as burns, were registered at the hospitals, instead of the code for the EI itself. This would lead to an underestimate of the associations in the study. It may also be the case that minor burns are not registered in relation to an EI if patients receive only one ICD-10 code.

For the part of the cohort drawn from the DWEA register, we have no reason to believe there would be any difference in reported exposure, even though the number of EIs diminished over time, which was the general trend for all occupational accidents in Denmark during the period studied. The severity and other characteristics of the EI were not registered, because the definition of an EI was based on the DNPR’s ICD-10 code, and exposure type of the injury in the DWEA register. Some previous studies have distinguished between low- and high-voltage injuries, indicating that high-voltage injuries were more frequently associated with chronic pain (Radulovic et al. 2019) and long-term symptoms such as pain (Rådman et al. 2016). As a proxy for injury severity, we carried out a sub-analysis restricted to persons hospitalized for at least 1 day, and results showed that the risk of *unspecified pain* was higher among the more severe injuries. The validity of using hospitalization as a proxy for severity is debatable, since length of hospitalization may not fully capture the severity of the injury, as for instance burn patients would probably be hospitalized longer than a patient with severe pain, despite a somewhat less severe injury. Thus, it would have been more transparent to stratify according to the type of injury. However, we did not have information on the type of injury and the severity from the registries. But as we applied length of hospitalization in a dichotomous manner with a cut-off at 1 day or more, it is used as a somewhat crude measure, where only the most trivial injuries are coded as minor. As the hospital stay after most of the EIs was short, we see this as a minor problem because all potential severe injuries were included in the group with a length of hospitalization more than 1 day. Furthermore, EI patients with (severe) burns would probably be diagnosed with an organ specific diagnosis and would not be diagnosed with an unspecific pain diagnosis.

A considerable number of EIs reported to the DWEA register either did not involve hospitalization, or involved only an outpatient visit. This is somewhat surprising, as the criterion for reporting an occupational accident to the DWEA register is that it results in sick leave at least the day after the accident, indicating some degree of severity. However, it is also possible that some of the injured persons were examined by their general practitioners, and, therefore, not registered in the DNPR. Another limitation may be that individuals who sustained an EI were not censored, if they were registered with more than one EI ICD-10 code during follow-up. We decided on this approach since it was not possible to distinguish between the consequences of the original injury or another new event.

Another limitation of this study was our choice of match controls. It was very difficult to identify the optimal type of injury to match an EI. EIs are very heterogeneous in their degree of severity, so we favoured a similarly heterogenetic injury group. It also had to involve a relatively frequent type of injury, for us to be able to find an adequate number of suitable match controls. Moreover, it was important that we use only matching diagnoses that were not believed to cause the outcomes in which we were interested. Therefore, we used three different types of match controls: controls with dislocations/sprains, controls with eye injuries, and controls engaged in the same type of work as the individuals who sustained EIs. The drawback of matches 1 and 2 was that the injuries were not as severe as an EI may be. This could cause us to overestimate the frequency of the outcomes in which we were interested. However, those match controls shared the characteristics that they were also examined for an injury at a hospital within the same year and, therefore, sought the same level of healthcare as the EI individuals.

For match 3 we used controls with the same occupation. However, the drawback of this was that these individuals did not have registered injuries, and were probably not in the health care system at the time of the match. Thus, our results for this match may be somewhat overestimated if EI individuals sought another level of healthcare than the match controls. In that case, the bias would not be removed by adjusting for the length of hospitalization, because this was not available for the match controls. A similar approach was applied to a previous Danish cohort study of cardiac disease and mortality, where random controls from the general population were matched with individuals who sustained EIs, using age and gender (Hansen et al. 2017). We aimed to match with other injured individuals (matches 1 and 2) to try to avoid overly healthy controls, and also took socioeconomic position into account in the occupation match (match 3).

The size of this study was the largest possible, using Danish data. As we decided to include 2 years of observation prior to an accident, to exclude individuals with the outcome in which we were interested, and 2 years of observation for new outcomes to appear, this limited us to 19 years, from 1996 to 2014.

The results of this study may be generalized to populations with access to hospital treatment and/or a system for registering work injuries that is comparable to that found in Denmark.

## Conclusion

This study confirms that exposure to EIs increases the risk of being diagnosed with *unspecified pain* or *unspecified soft tissue disorders* both at short and long terms. Our results also indicated that the risk of *unspecified pain* as sequela is related to the severity of the injury.

Our findings call for further studies to disentangle the complex mechanisms between exposure to electrical current and unspecified pain, to improve clinical praxis.

## Data Availability

All data were stored and processed in a secure, protected server at Statistics Denmark. Access to data can be obtained by application to Statistics Denmark.
